# Employees’ Work-Related Well-Being during COVID-19 Pandemic: An Integrated Perspective of Technology Acceptance Model and JD-R Theory

**DOI:** 10.3390/ijerph182211888

**Published:** 2021-11-12

**Authors:** Marjan Shamsi, Tatiana Iakovleva, Espen Olsen, Richard P. Bagozzi

**Affiliations:** 1 Department of Innovation, Leadership, and Marketing, University of Stavanger, 4021 Stavanger, Norway; tatiana.a.iakovleva@uis.no (T.I.); espen.olsen@uis.no (E.O.); 2 Ross School of Business, University of Michigan, Ann Arbor, MI 48109-1234, USA; bagozzi@umich.edu

**Keywords:** COVID-19, remote working, technology acceptance, well-being, work engagement, perceived team support, mental load

## Abstract

Employees’ work-related well-being has become one of the most significant interests of researchers and organizations due to the COVID-19 pandemic. This study examines how job characteristics such as mental load and team support, and technology-related factors such as perceived ease of use, perceived usefulness, and technology acceptance, impact employees’ work engagement as a dimension of work well-being. Data were collected through a sample of 610 academic employees from three Norwegian universities after COVID-19 restrictions were implemented. The structural model estimation showed that mental load, perceived team support, and technology acceptance were significantly related to work engagement. It also showed that perceived usefulness, perceived ease of use, and mental load were significantly related to technology acceptance. Furthermore, the analysis showed that technology acceptance partially mediates the relationship between job characteristics and work engagement, and fully mediates the relationship between technology-related perceptions and work engagement. Building on the technology acceptance model (TAM) and job demands-resources (JD-R) theory, this study provides insights into the effects of job-related and technology-related factors on remote workers’ well-being. By doing so, we contribute to the existing literature by demonstrating how remote working with the use of newly implemented technologies can be related to employees’ well-being during a pandemic.

## 1. Introduction

During earlier centuries, different pandemics have hit several countries worldwide and had health, social, cultural, and economic consequences for societies [1,2]. The most recent pandemic, which emerged in December 2019, was the outbreak of coronavirus disease (COVID-19) that infected millions of people worldwide in early 2020 [3]. The rapid spread of COVID-19 within a short time affected various aspects of human life and caused a new crisis in the world, convincing people of its long-term effects on people’s physical health, mental health, and well-being [4,5,6]. As a result, different countries worldwide have enacted various rules since the beginning of the pandemic to prevent the transmission of this infectious disease and its devastating consequences. Norway was one country that adopted sectoral measures, and the government directed employers and employees to work from home. Affected by these measures, many Norwegian organizations decided to start remote working and reduce people’s face-to-face activities as much as possible.

The COVID-19 situation has provoked a workplace environment change and has triggered digitalization in Norwegian organizations by forcing most employees to work remotely and use current or new digital communication tools to meet their communication needs. Remote working refers to “a flexible work arrangement whereby workers work in locations, remote from their central offices or production facilities, the worker has no personal contact with co-workers there, but is able to communicate with them using technology” [7] (p. 530). This work mode could pose new opportunities and challenges to organizations and their members. Initial studies that have focused on the impact of remote working through different technologies on employees and employers’ experiences have found that it could cause borderless working, where the work world overlaps other aspects of life and leads to work–home interference [8,9]. Other negative implications of remote working include lower chances of workers’ promotion [10], burnout [11], cognitive stress complaints [11], and loneliness [9]. Despite these drawbacks, teleworking offers many advantages for both employers and employees, such as higher job performance [10], job satisfaction [10,12,13], organizational commitment [8,13], engagement and enthusiasm in work [8,11], lower job turnover [10], reduced work strain during time pressure [14], decreased costs [15], and saving time and resources [8,10,15]. 

Among the mentioned outcomes of remote working, perhaps the most crucial consequence for organizations during the COVID-19 crisis is employees’ work-related well-being (See [16]). In fact, employees could experience different challenges and opportunities in confronting this unexpected change, which in turn could affect their well-being [17]. For example, Wang, Liu, Qian, and Parker [9] focused on the impact of virtual job characteristics and individual differences in remote workers’ experienced challenges during the COVID-19 situation and found that employees who received more social support from work during remote working experienced higher levels of well-being. They also showed that employees with higher workloads due to remote working experienced lower levels of well-being. Other researchers have shown that the extent or intensity of telecommuting by employees could indirectly affect their well-being (i.e., burnout, work engagement, and cognitive stress complaints) through mediating job characteristics such as social support from colleagues, participation in decision-making, task autonomy, and work–family conflict [11]. Our study examines the effect of two virtual job characteristics on employees’ well-being, namely mental load and perceived team support, often considered as job demands and resources in the job demands-resources (JD-R) theory [18].

In addition, organizations introduced employees to new technologies to perform their tasks with the pandemic onset. The literature shows that implementing new technologies fundamentally affects individuals (e.g., [19]) by forcing them to develop task-related digital competencies, evaluate different new digital communication tools, and adopt the most efficient digital communication tools [19,20,21,22]. Accordingly, acceptance and use of new technologies required employees to evaluate the various tools introduced during remote working due to COVID-19 restrictions. Based on the technology acceptance model (TAM) developed by Davis [23,24,25], there are two major constructs, perceived ease of use (PEOU) and perceived usefulness (PU), which ascertain attitudes toward the technology and actual use of it [24,25]. TAM literature shows that PEOU and PU have positive associations with technology acceptance [24,26]. Based on this literature, in this study, we assume that PEOU and PU are positively related to technology acceptance. In addition, our study extends the TAM by adding conditions under which employees decide to use newly introduced technologies while working from home. We expect that when employees perceive more support from their teams or face less mental load when accomplishing job tasks, use of technology will increase.

Also, in contrast to previous research on employees’ well-being, which primarily focuses on the negative side of technology use (e.g., [21,22,27,28]), this research aims to confirm a few other studies that focus on the positive effect of technology use on well-being (e.g., [14,21]). Hence, we assume that acceptance of new digital technologies can be positively associated with work-related well-being. Particularly, we argue that this relationship is more likely to become positive in the COVID-19 situation since many employees had no choice but to work remotely to communicate with their co-workers and customers. Therefore, digital technology has provided them with an opportunity to interact with others and perform their job tasks while working from home, which in turn may result in higher work-related well-being. We also attempt to illuminate the relationship between technology use and the well-being of employees by addressing this relationship during COVID-19 remote working. In other words, while several studies so far have investigated the impact of using technologies on office workers’ well-being [27,29,30,31], to our knowledge, few studies have explored how technology use during COVID-19 remote working is related to employees’ health and well-being. Molino et al. [32] demonstrated the risk factors for behavioral stress among Italian workers using different technologies during COVID-19. Other researchers have considered the influence of organizational communication using technologies on psycho-physical disorders during the COVID-19 pandemic [33]. Nevertheless, organizational researchers have not yet thoroughly examined how acceptance of new technologies has impacted employees’ work-related well-being during COVID-19 remote working.

Finally, this study investigates the role of technology acceptance as an underlying mechanism in the relationship of virtual job characteristics (mental load and perceived team support) and technology-related perceptions (perceived ease of use and perceived usefulness) with employees’ work-related well-being. Our study goes beyond the JD-R theory by introducing TAM’s constructs to specify the conditions under which employees’ perceptions regarding job and technology lead to their well-being. In this study, well-being at work is depicted as work engagement, a positive cognitive-affective condition characterized by vigor, dedication, and absorption [34]. 

**Figure 1:**
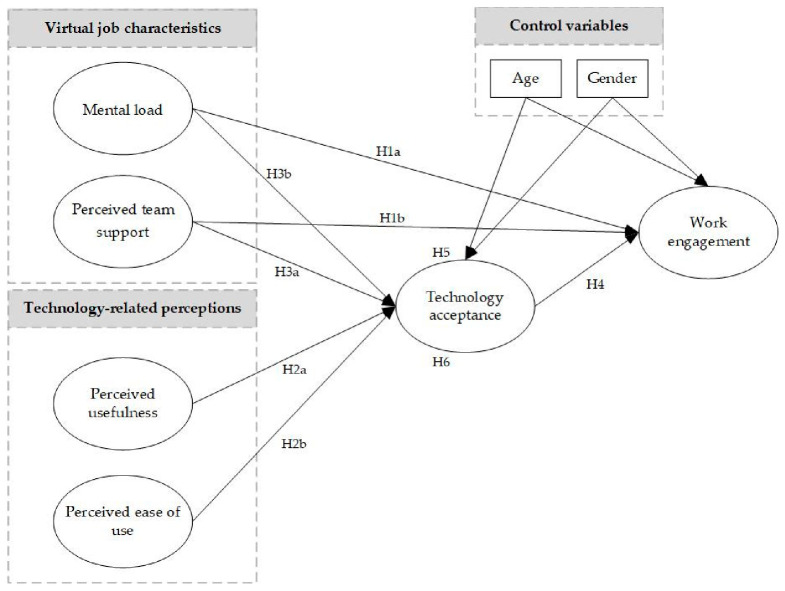
The conceptual framework of the study.

To sum up, our study makes four important contributions to organizational psychology research. Firstly, we provide a theoretical model that integrates two substantial models to predict employees’ well-being during remote working: the JD-R model and TAM. This research model has not been systematically examined before. In doing so, we evaluate the effects of two different sets of constructs, both job-related and technology-related concepts, on employees’ work engagement. We argue that virtual job characteristics and users’ perceptions will increase work engagement through users’ technology acceptance during remote working in the COVID-19 situation. Secondly, we add to the JD-R theory by examining the direct effect of virtual job demand (i.e., mental load), a virtual job resource (i.e., perceived team support), and users’ technology acceptance on employees’ work engagement during remote working. Thirdly, we extend TAM to identify antecedents of users’ technology acceptance. More precisely, we add mental load and perceived team support to TAM to explore how these concepts affect technology acceptance. Finally, we explore the mediating role that technology acceptance has in the structural equation model. Figure 1 summarizes the mediating mechanism linking virtual job characteristics and technology-related perceptions to work engagement.

### 1.1. Work Engagement 

Organizational psychologists and organization researchers have long referred to work engagement as a component of work-related well-being. For instance, Warr, a well-known researcher in occupational psychology, proposed a work-related well-being model in which work engagement comprises part of one of the model dimensions. This model is characterized by three dimensions: pleasure-displeasure, anxiety-comfort, and enthusiasm-depression [35]. Fatigue-vigor has been suggested as a fourth dimension [36]. Pleasure-displeasure indicates a person’s job satisfaction level. The anxiety-comfort dimension demonstrates that a combination of low pleasure and high mental arousal in a person causes anxiety; in contrast, low levels of mental arousal and pleasure result in comfort. The enthusiasm-depression dimension reveals that people who experience low levels of pleasure and mental arousal will feel depression, while those who encounter high levels of pleasure and mental arousal tend to feel more enthusiasm. Work engagement is a component of the third dimension [35]. 

Work engagement is defined as “a positive, fulfilling, work-related state of mind that is characterized by vigor, dedication, and absorption” [34] (p. 295). In this definition, work engagement is a persistent and pervasive affective-cognitive state negatively associated with burnout. In other words, people with high work engagement experience fulfillment, while those with high burnout will feel empty [37]. Vigor could be experienced in workers with high energy levels and mental resilience when working, a passion for devoting effort to work, and persistence even in facing problems. Dedication could be observed in people who are strongly involved in their work; feel a sense of significance, inspiration, and enthusiasm; and consider work tasks as a challenge. Absorption refers to being immersed and entirely concentrated in work while time passes quickly [37].

The importance of improving work engagement in organizations has been investigated in previous studies. The literature shows that engaged employees are often highly energetic individuals with higher job performance, organizational commitment, positive job-related attitudes, health, and well-being, and lower absence rate and intention to leave the organization than non-engaged workers [34,38]. Engaged employees also have a positive attitude; they are able to create their own job and personal resources, transfer their engagement to others, and change their work environment [37,38,39]. They may feel tired after working hard, but they perceive their tiredness as a pleasant experience due to its association with positive achievements [37]. These findings show that employees’ work engagement is an essential concept in organizations that has positive consequences at the individual and organizational levels, and it can be improved by applying particular human resource management strategies, focusing on increasing employees’ motivation, challenging them, and encouraging their learning and development at work [38].

#### Job Characteristics and Work Engagement

So far, antecedents of engagement have mainly been investigated in several studies focused on work engagement within the framework of the JD-R model [18]. According to the JD-R model, two sets of working conditions (job characteristics) predict employees’ well-being in different occupational groups: job demands and job resources (e.g., [34,40,41]). Job demands refer to “those physical, social, or organizational aspects of the job that require sustained physical and/or psychological (i.e., cognitive and emotional) effort on the part of the employee, and are therefore associated with certain physiological and/or psychological costs” [40] (p. 501). This set of job characteristics probably elicits strain, but they are not essentially negative; they may act as job stressors when an employee has to put significant effort into meeting required demands and may evoke such negative responses as burnout [34]. On the other hand, job resources are defined as “those physical, psychological, social, or organizational aspects of the job that may (a) reduce job demands and the associated physiological and psychological costs, (b) are functional in achieving work goals, and (c) stimulate personal growth, learning, and development” [40] (p. 501). The JD-R model proposes a fundamental assumption in which the combination of high job demands (e.g., mental load) and lack of resources (e.g., lack of social support in the workplace) may increase burnout and reduce work engagement [34,40,41]. Furthermore, a combination of high job resources and either high or low levels of job demands may increase work engagement [18]. 

It can be concluded from the JD-R model that job resources lead to work engagement and job demands result in burnout. Still, previous findings have shown that the relationship between job demands and work engagement is ambiguous. Although some studies have found no relationship between job demands and engagement, other studies have found either positive or negative relationships between these constructs [42]. Based on these previous findings, Crawford, LePine, and Rich [42], in a meta-analysis, suggested a challenge-hindrance stressor framework rooted in transactional stress theory [43]. According to this framework, some job demands that have been classified as challenge stressors result in increased work engagement. These types of demands are perceived as opportunities that will result in employees’ learning, achievement, personal growth, and future gains. Examples of challenge stressors are time pressure, workload, and job responsibility. The other job demands, called hindrance stressors, lead to decreased work engagement. Employees perceive these stressful demands as constraints or barriers that might prevent their personal growth and goal attainment. Examples of such stressors are role conflict, role ambiguity, and organizational politics [42].

Building on the JD-R model in conjunction with the challenge-hindrance stressor framework, we argue that high mental load and perceived team support as two virtual job characteristics in the COVID-19 situation [9] will increase employees’ work engagement. 

Mental load (sometimes referred to as mental workload or cognitive load) is defined as the extent to which a job requires one’s attention and concentration [44]. Mental load acts as a cognitive job demand that influences the brain’s functions involved in information processing [45,46] and requires a person’s mental work or effort to perform the task(s). Since each individual’s information processing capacity is limited, facing multiple tasks that require mental concentration may result in mental workload [47]. In other words, the mental load may be a result of task intensity. Literature shows that individuals with considerable mental workload will suffer from high levels of work-related stress and low levels of job performance [48]. In other words, when accomplishing the main tasks is difficult for employees, their mental processing will increase, leading to high job stress and low job and organizational performance, if processing demands for tasks exceed a person’s capacity for information processing [48]. Despite studies finding a negative relationship between mental load and work-related outcomes [48,49], some studies have found a positive association between this job demand and work outcomes such as work engagement. For example, Verbruggen [50] found positive relationships between mental load and two work engagement dimensions, vigor and dedication. This researcher posits that working with a lot of information and being mentally occupied may lead to more persistence and enthusiasm in employees. D’Emiljo and Du Preez found in a study of nursing practitioners that mental load is positively related to work engagement [51]. Pace and Sciotto [52] also reported a positive relationship between mental load and dedication and absorption in work in Italian fixed-term researchers.

Based on the challenge-hindrance stressor framework and existing literature, our study assumes that task and effort intensification, which might result from digitalization and fast technological changes [53,54] due to the COVID-19 pandemic, could lead to mental load, which in turn could promote employees’ work well-being. We argue that remote working using new technologies is a challenging situation that increases employees’ mental load and promotes their work engagement, because employees believe that their invested time and energy will be rewarded by personal growth or goal achievement. Therefore, we posit that mental load is an immediate positive determinant of work engagement among employees working from home during the COVID-19 outbreak. This leads to the following hypothesis:

**Hypothesis** **1a.** *Mental load has a positive relationship with work engagement during remote working*.

Several studies so far have revealed that job resources, such as perceived support from the organization, supervisor, and colleagues, can positively predict work engagement [55,56]. Our study focuses on perceived support from the organization’s departments and considers each department a work team. Bishop, Scott, and Burroughs [57] define perceived team support (PTS) based on the definition of perceived organizational support (POS) [58]. According to this definition, PTS is “the degree to which employees believe that the team values their contribution and cares for their well-being” [57] (p. 1114). Previous research shows that perceived support from co-workers and superiors fosters work engagement through a motivational process [59]. In other words, employees who receive more support from others are likely to be more willing to dedicate their efforts and energies to their job tasks, resulting in higher work engagement [55,56]. Wang et al. [9] also explained that social support is a virtual job characteristic in the COVID-19 situation that affects employees’ well-being and performance while working from home. Altogether, we propose that perceived team support as a virtual job characteristic increases employees’ work engagement during remote working. Thus, we hypothesize:

**Hypothesis** **1b.** *Perceived team support has a positive relationship with work engagement during remote working*.

### 1.2. Technology Acceptance: Antecedents and Work Engagement 

Research in technology acceptance was initiated in the 1970s with emerging technology needs and organizations’ failure to introduce new systems [60]. Since then, researchers have proposed several theories and approaches to address and predict factors that make people accept and use new information systems (IS) [61,62]. The TAM proposed by Davis [25] in 1989 is one such approach tested and extended frequently by other researchers [62]. Based on the theory of reasoned action (TRA) [63] that attempts to explain and predict human behavior, Davis proposed the TAM to show why a potential user will accept or reject the use of information technology [24,61,62]. Based on TRA, TAM proposes that PEOU and PU are two primary predictors of users’ attitudes (A) and behavioral intentions (BI), which have been assumed to have a strong association with the users’ actual computer usage behavior [24,25,61]. Computer usage is determined by BI in this model; BI is jointly determined by A and PU; and A is jointly influenced by PU and PEOU, with relative weights estimated by regression [24]. TAM also claims that PEOU and PU can be influenced by external variables such as system characteristics and features [24].

#### 1.2.1. Technology-Related Perceptions and Technology Acceptance

PEOU is defined as ”the degree to which the prospective user expects the target system to be free of effort” [24] (p. 985). PU refers to “the prospective user’s subjective probability that using a specific application system will increase his or her job performance within an organizational context” [24] (p. 985). PU and PEOU are expected to be fairly general determinants of user technology acceptance [24]. Over the past few decades, psychologists and IS researchers have replicated Davis’ study [25] to contribute empirical evidence on the association between PU, PEOU, and actual use [24,61,62,64]; however, investigating the direct effects of these two particular user beliefs on technology acceptance behavior, specifically during remote working, is a different approach which few studies have used (e.g., [65,66,67]). Consistent with extant research, we argue that when employees work from home due to the COVID-19 pandemic, their perceptions of using new technology will influence their decision to use it. Since the TAM literature posits that PEOU and PU are two important determinants of actual use, we expect that employees who find technology useful and easy to use will be more likely to use it. Therefore, we hypothesize:

**Hypothesis** **2a** . *Perceived usefulness of digital communication tools has a positive relationship with technology acceptance during remote working*.

**Hypothesis** **2b.** *Perceived ease of use of digital communication tools has a positive relationship with technology acceptance during remote working*.

#### 1.2.2. Job Characteristics and Technology Acceptance

Several studies have extended the TAM to better understand why people decide to accept or reject new technology. These researchers have used the TAM framework and have added new constructs and relationships, specifically beyond PU and PEOU, to describe user acceptance [68]. Examples of these external constructs are subjective norms, perceived behavioral control [69], result demonstrability, image, personal innovativeness [70], social influence, cognitive instrumental processes [23], trust, and perceived risk [71]. 

Another TAM variation is the unified theory of acceptance and use of technology (UTAUT), which suggests that performance expectancy, effort expectancy, facilitating conditions, and social influence are four direct determinants of user acceptance and usage behavior [72]. Performance expectancy refers to “the degree to which an individual believes that using the system will help him or her to attain gains in job performance” [72] (p. 447). It reflects five constructs from other models, including perceived usefulness, extrinsic motivation, job fit, relative advantage, and outcome expectation. Effort expectancy is defined as “the degree of ease associated with the use of the system” [72] (p. 450); three constructs, including perceived ease of use, complexity, and ease of use, are related to this concept. Social influence refers to “the degree to which an individual perceives that important others believe he or she should use the new system” [72] (p. 451) and captures three constructs, including subjective norm, social factors, and image. Facilitating conditions refer to “the degree to which an individual believes that an organizational and technical infrastructure exists to support use of the system” [72] (p. 453) and is represented in other models as perceived behavioral control, facilitating conditions, and compatibility. Facilitating conditions address the technological and/or organizational aspects of the environment and are supposed to eliminate usage barriers and directly affect acceptance of a system [72]. This concept is compatible with the construct of perceived user resources added to TAM by Mathieson, Peacock, and Chin [73]. These authors defined perceived user resources as “the extent to which an individual believes that he or she has the personal and organizational resources needed to use an IS” [73] (p. 89). Perceived user resources are an attribute of both the system and the user’s environment; in other words, this attribute focuses on users’ perceptions of the technology and the resources (e.g., support from others), which can promote or prevent their acceptance behavior [72]. Based on this literature, we focus on the relationship of remote workers’ perceived team support, as a user’s resource, with technology acceptance. Thus, we hypothesize:

**Hypothesis** **3a.** *Perceived team support has a positive relationship with technology acceptance during remote working*.

In a recent extension of UTAUT, Dang et al. [74] added two constructs to this model: mental workload and task-technology fit as predictors of technology acceptance. These researchers showed that mental workload and task-technology fit could significantly predict users’ acceptance of social media search systems. They argued that perceived high cognitive load would reduce use of a system by negatively influencing performance expectancy, effort expectancy, and facilitating conditions. 

Generally, the literature on cognitive load (e.g., [75]) suggests that due to people’s limited cognitive resources, introducing new information (e.g., a new tool) results in cognitive load, which will consequently interfere with task performance and satisfaction with the tool [76,77]. In other words, if a tool is easy to use and requires fewer cognitive resources, people are more likely to use it to accomplish the task than if it is hard to use [76]. Schmutz et al. [77] also revealed that cognitive load negatively influences user satisfaction among people using the websites of four e-commerce systems. The authors posited that when the perceived cognitive load on a person using a system to perform the task is high, he/she will feel less satisfied with the system’s functionality and use. Based on these findings, our study argues that since people have used newly implemented technologies to perform their job tasks and communicate with others while working from home during the COVID-19 pandemic, they will have experienced more mental load than before, and experiencing more mental load due to using new technologies will influence their acceptance of these tools. Therefore, we hypothesize:

**Hypothesis** **3b.** *Mental load has a negative relationship with technology acceptance during remote working*.

#### 1.2.3. Technology Acceptance and Work Engagement

With the emergence of the coronavirus crisis, working in a remote mode became one of organizations’ most common working methods. Research shows that growing remote working engenders technological changes in organizations exposed to it, and technological changes result in more people’s technology usage and the introduction of new opportunities (i.e., greater flexibility and reactivity) and challenges (i.e., increased complexity and changing customer preferences) [8,54]. Based on the JD-R model, which suggests that job conditions may act as demands or resources [40], using technology for job-related activities can serve as a job demand or resource [78]. Building on this framework, previous studies have revealed two main research trends regarding the effect of technology use on work-related outcomes such as well-being. The first trend has addressed technology adoption as a perceived demand with adverse effects on the well-being of employees working from home or the office (e.g., [21,22,27,28,29,30,78]). According to this viewpoint, technology acceptance may act as a job demand if it increases workload, job pressure, and effort (see [78]). The second trend has viewed technology as a resource with positive effects on employees’ well-being [14,21,28,30,78]. This point of view posits that technology may act as a job resource by providing support for effective communication, interaction, and flexibility in location [78]. For example, Ter Hoeven et al. [21] revealed that communication technology use would influence employees’ well-being by producing a specific set of resources (efficient communication and accessibility) and demands (interruptions and unpredictability). Molino et al. [31] also showed that technology acceptance positively increases the work engagement of white- and blue-collar workers. These researchers argued that technology acceptance as a resource will increase employees’ work engagement by fostering the motivational process. 

Previous studies provide evidence to understand the relationship between technology use and employees’ well-being within the JD-R framework. Since none of these studies were conducted during the COVID-19 pandemic when most employees were forced to work remotely, there is still a lack of evidence for understanding this relationship. Therefore, it is necessary to study how technology acceptance has affected employees’ well-being during the Coronavirus lockdown. To better understand the effects of technology acceptance within the JD-R model context, we focused on work engagement as a well-being dimension and a psychological outcome generally associated with the JD-R model [38]. Based on previous studies and COVID-19 remote working challenges, we argue that technology acceptance as a resource has created significant value for organizations by motivating employees to perform their work beyond the usual physical workplace during the COVID-19 pandemic. In other words, it is expected that technology acceptance activates motivational outcomes such as work engagement in employees who have to work from home due to restrictions. Although this hypothesis replicates Molino et al.’s [31] finding, we note the value of examining this relationship among employees working remotely due to the COVID-19 crisis. Therefore, we posit that:

**Hypothesis** **4.** *Technology acceptance has a positive relationship with work engagement during remote working*.

### 1.3. The Mediating Role of Technology Acceptance

Various studies have suggested a direct relationship of perceived support and mental load with work engagement. Our research model views technology acceptance as the mediating mechanism relating perceived team support and mental load to work engagement. That is, employees with higher perceived team support and lower mental load during remote working are more likely to be engaged in their work because they are accepting and using technologies that may facilitate the accomplishment of their job tasks, communication with their colleagues, and fulfillment of their role when they cannot be present in the workplace. As reviewed earlier, Molino et al. [31] found support for the mediating role of technology acceptance in the relationship between personal and organizational antecedents and work engagement. Based on the Worker-Centric Design and Evaluation Framework for Operator 4.0 [79] and the motivational process of the JD-R model [41], these researchers argue that resilience, goal orientation, and opportunities for information and training might increase employees’ work engagement through employees’ experience with a new tool [31]. Building on the JD-R model and previous studies, we assume that technology acceptance is a mediator of the virtual job characteristics-work engagement relationship. Therefore, we hypothesize:

**Hypothesis** **5.** *Technology acceptance partially mediates the relationships between work engagement and (a) mental load and (b) perceived team support*.

We also expect technology-related perceptions (i.e., PEOU and PU) to increase work engagement through technology acceptance. Although no previous study has been found to specifically investigate the causal relationship of PEOU and PU with work engagement, we argue that when a remote worker finds a new tool easy to use and useful in accomplishing job tasks, he/she is more likely to accept it as a job resource. Under this circumstance, the user will show a willingness to use technology, which in turn will facilitate his/her work motivation and engagement by providing an opportunity to achieve his/her work goals. Therefore, technology acceptance can strengthen the association of PEOU and PU with work engagement. Thus, we hypothesize:

**Hypothesis** **6** **.** *Technology acceptance fully mediates the relationships between work engagement and (a) perceived usefulness and (b) perceived ease of use*.

## 2. Materials and Methods

### 2.1. Respondents and Procedures

A cross-sectional survey was used to analyze the primary research hypotheses. The survey was distributed to the 3140 academic employees in three universities in Norway. Respondents were employees who worked remotely using recently implemented digital communication technologies during their day-to-day work for teaching, research, and dissemination of knowledge after COVID-19 restrictions were implemented on 15 March 2020. To gain permission to conduct the study in these three universities, we contacted top management board members of each university to explain the project’s purpose and request approval to distribute the survey. When our request to conduct an online survey of scientific employees was approved, departments responsible for communication in each university published an announcement on the university’s internal webpage one week before the initial survey period, explaining the survey information and purpose. One week later, an e-mail was sent to academic employees that included a link to the online survey. The data were collected through the Qualtrics web system from 18 July to 15 October 2020. The overall response rate was 23.6%, and 610 respondents remained in the final sample after data cleaning

Although some participants partially returned to their office during the data collection period, they were communicating through technologies since the government encouraged all employees to work from home as much as possible. Furthermore, we asked them to express their working experiences during the lockdown because we focused on particular aspects of remote working experience due to COVID-19 restrictions, such as using newly introduced digital technologies. Therefore, participants indicated their opinion considering their experiences during the COVID-19 lockdown.

The general characteristics of the respondents are presented in Table 1.

### 2.2. Measures

We used items from established scales to measure the study variables shown in Figure 1. A Norwegian translation company translated the English language measures into Norwegian, and a Norwegian-speaking professor reviewed and edited the translated survey. To confirm the questionnaire’s accuracy, we translated it back into English. Then it was refined by a bilingual English-Norwegian speaker. 

Work engagement was measured with the three-item version of the Utrecht Work Engagement Scale (UWES-3) [80]. A sample item from the UWES-3 scale is “At my work, I feel bursting with energy”. Participants responded on a five-point scale ranging from strongly disagree (1) to strongly agree (5). 

Technology acceptance was assessed by three items adapted from the user satisfaction scale [81]. A sample item is “I am satisfied with the performance of these digital tools”. Participants responded on a five-point scale ranging from strongly disagree (1) to strongly agree (5). 

Perceived usefulness (PU) was measured with three items adapted from Davis [25]. A sample item is “Using these digital communication tools will improve my performance in my job”. Participants responded on a five-point scale ranging from strongly disagree (1) to strongly agree (5). 

Perceived ease of use (PEOU) was assessed using scales adapted from Davis [25]. A sample item is “My interaction with these digital communication tools is clear and understandable”. Participants responded on a five-point scale ranging from strongly disagree (1) to strongly agree (5).

Mental load was measured with four items adapted from the English version of the French multidimensional measure of job demands and resources, namely the Questionnaire sur les Ressources et Contraintes Professionnelles (QRCP) [82]. A sample item is “I have to give continuous attention to my work”. Participants responded on a five-point scale ranging from strongly disagree (1) to strongly agree (5). 

Perceived team support was assessed using a four-item scale adapted from the measure of perceived organizational support [58]. We re-worded items from the original scale by replacing the word “organization” with the word “department” to focus on the departments’ situation as participants’ workgroup. A sample item from this scale is “The department cares about my general satisfaction at work”. Participants responded on a five-point scale ranging from strongly disagree (1) to strongly agree (5). 

Control variables in this study were two demographic variables, gender and age, which were measured as categorical variables.

### 2.3. Data Analysis

Descriptive statistics, including means and standard deviations, were performed to explore the data. To assess the measuring instruments’ internal consistency, Cronbach alpha coefficients (α) were employed [83]. Pearson product-moment correlation coefficients were conducted to indicate the relationships between variables. To test the analyses mentioned above, the commercial software IBM SPSS Statistics 26 (IBM Corporation, Armonk, NY, USA) was employed. AMOS 26.0 (IBM Corporation, Armonk, NY, USA). [84] was used to evaluate confirmatory factor analysis (CFA) and structural equation modeling (SEM).

Several steps were conducted to handle potential measurement bias and to avoid potential multicollinearity issues. To evaluate the measurement model, the CFA was carried out and the internal consistency, convergent validity, and discriminant validity of the study constructs were tested. We used Cronbach’s alpha (>0.7) [85], composite reliability (CR > 0.6), and average variance extracted (AVE > 0.5) [86] to evaluate the internal consistency of constructs (Table 2). We tested the CFA model and factor loadings (>0.5) to assess the convergent validity of constructs [87]. Also, we assessed discriminant validity using the Fornell-Larcker criterion [88]. According to this criterion, discriminant validity is achieved if none of the square roots of AVE values is more than the correlation of the latent variables.

SEM methods were employed to test the research model shown in Figure 1 and served to test hypotheses 1–6. To assess the model’s goodness of fit, four recommended practical model fit indices were employed [86]: root mean square error of approximation (RMSEA), Tucker and Lewis index (TLI), comparative fit index (CFI), and standardized root mean square residual (SRMR). Recommended cutoff values for indexes are RMSEA ≤ 0.06, TLI ≥ 0.95, CFI ≥ 0.95, and SRMR ≤ 0.08 [89,90]. Since Chi-square (χ^2^) is sensitive to sample size, it was not employed to assess model fit as recommended [86]. Furthermore, in SEM analysis, we controlled for gender and age. Bootstrapping has been employed to test the significance of the indirect effects by extracting 2000 bootstrap samples from the original data [91]. therefore, it was used to test H5 and H6 in the model.

Since the measurement model is integrated in the structural model, the above-mentioned procedures adequately document satisfactory validity and reliability of this study.

## 3. Results

### 3.1. Measurement Model Evaluation

In testing the internal consistency of measures, results show that all Cronbach’s alpha values ranged between 0.75 and 0.92, higher than the recommended level of 0.7 [85]. Also, as Table 2 shows, the CRs range from 0.74 to 0.92, and the AVEs from 0.50 to 0.81, which is compatible with the recommended levels of these indicators [86]. Therefore, the results support satisfactory internal consistency of the study constructs.

We also assessed convergent and discriminant validity, which must be considered as two key constituents in model evaluation (e.g., [92]). Therefore, CFA was performed to evaluate the scales’ convergent validity. We built a CFA model with six latent constructs; results showed the CFA model fitted the data well. The goodness-of-fit statistics for the model are as follows: RMSEA = 0.06, TLI = 0.92, CFI = 0.94, and SRMR = 0.05. The results also show that all items’ factor loadings are acceptable (Table 2). These results indicate the satisfactory convergent validity of the model.

To assess discriminant validity, we examined correlation for all the study variables using the Fornell-Larcker criterion [88]; the correlation matrix revealed that variables show low to moderately high correlation. Table 3 demonstrates that all correlations between constructs are lower than the squared root of AVE, which shows discriminant validity was achieved for all constructs.

### 3.2. Structural Model Evaluation

The hypothesized model, in which technology acceptance was a mediator between perceived team support, mental load, PU, PEOU, and work engagement showed a good fit to the data. The goodness-of-fit indices for the model are as follows: RMSEA = 0.060, TLI = 0.91, CFI = 0.93, SRMR = 0.05.

**Figure 2:**
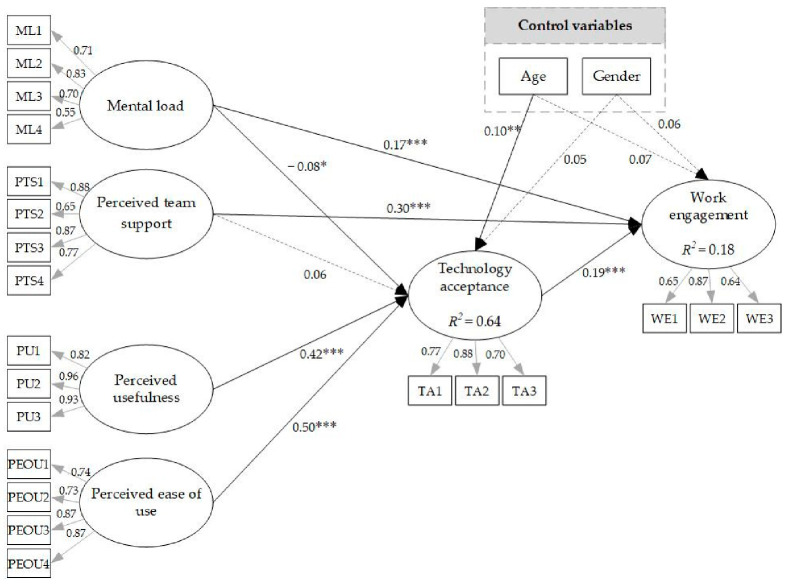
The final model. Note: Discontinuous lines mean nonsignificant relationships. *** *p* < 0.001; ** *p* < 0.01; * *p* < 0.05.

According to the model (Figure 2), mental load (H1a: β = 0.17, *p* < 0.001) and perceived team support (H1b: β = 0.30, *p* < 0.001) were positively related to work engagement. Perceived usefulness (H2a: β = 0.42, *p* < 0.001) and perceived ease of use (H2b: β = 0.50, *p* < 0.001) were positively related to technology acceptance, and mental load was negatively related to technology acceptance (H3b: β = −0.08, *p* < 0.050). There was no significant relationship between perceived team support and technology acceptance (H3a). Technology acceptance also had a positive association with work engagement (H4: β = 0.19, *p* < 0.001). In addition, the model explained 64 percent of the variance for technology acceptance and 18 percent for work engagement.

### 3.3. Tests of Mediation Hypotheses

Table 4 shows the bootstrapping result for indirect effects. The results show a significant and negative indirect relationship between mental load and work engagement through technology acceptance (H5a: β = −0.020, CI 95% = −0.044, −0.006). Furthermore, the results show a significant and positive indirect effect from perceived team support to work engagement, mediated by technology acceptance (H5b: β = 0.008, CI 95% = 0.001, 0.019). Moreover, results indicate a significant and positive indirect effect from perceived usefulness (H6a: β = 0.074, CI 95% = 0.041, 0.118) and perceived ease of use (H6b: β = 0.042, CI 95% = 0.022, 0.067) to work engagement via technology acceptance. 

### 3.4. Control Variables 

The control variables, gender and age, were modeled. Results revealed that the only significant relationship was between age and technology acceptance (β = 0.10, *p* < 0.010). However, results showed that age and gender did not reduce the significant beta effects of all final model parameters. This result indicates that the control variables did not influence the overall findings.

## 4. Discussion

### 4.1. Interpretation of Findings and Theoretical Implications

The primary aim of this study was to explore the effect of job-related and technology-related factors on remote workers’ well-being. We employed two theories for that purpose: TAM and the JD-R model. In support of these two models, this study presented and examined a theoretical model on the relationships of virtual job characteristics, technology-related perceptions, and technology acceptance with the work-related well-being of employees using newly introduced technologies when working remotely due to the COVID-19 pandemic. This is the first study in the literature that integrates both job characteristics and technology-related constructs to explain employees’ work well-being. Moreover, few studies have examined the direct effects of job demands (i.e., mental load), job resources (i.e., perceived team support), and technology acceptance on work engagement drawing on the JD-R model. This study also adds to TAM literature by examining the direct effect of mental load and perceived team support on users’ technology acceptance. Finally, the study aimed to extend the literature on technology use and its implications for employees’ health and well-being. Likewise, from a mediation approach, this study attempted to understand how lower mental load, higher team support, and technology-related perceptions increase employees’ intention to reuse a technology, which will be reflected in their work engagement during remote working. This study’s results provide support for the research model. Findings will be discussed in this section.

Results regarding the predictor role of mental load (job demand) in work-related well-being (H1a) show a significant positive relationship between mental load and work engagement. This finding aligns with the challenge-hindrance stressor model and prior research [50,51,52]. This finding shows that mental load acted as a positive job demand during remote working, resulting in increased work engagement. According to the challenge-hindrance stressor model, cognitive demands at work, as challenging stressors, could be positively related to employees’ motivation and well-being [93]. The mental load might also serve as a practical challenge for employees, improving work engagement. This result confirms that when employees are experiencing a challenging situation like remote working in a pandemic, they will find meaning in mental load as a remote working challenge, making them more willing to invest time and energy to meet those challenges and achieve their work goals. Specifically, we argue that academic employees at universities are more likely to respond positively to challenging demands since they are interested in learning and achievement opportunities, which will result in their willingness to meet challenges and be reflected in greater work engagement. In addition, it is noticeable that previous research shows a curvilinear relationship between job demands and work engagement [94]. Therefore, the relationship between mental load, as a cognitive demand, and employees’ work engagement might be curvilinear across a fuller range of situations. In other words, work engagement will be at a lower level when the mental load is low, and as mental load increases, employees will adjust their motivation by becoming more engaged in their work. However, at some point, the relationship might level off or even decline, such that excessive mental load will lead to exhaustion and disengagement.

Also, results confirm hypothesis 1b, which assumed a positive direct relationship between perceived team support and work engagement. This finding emphasizes the motivational process assumption of the JD-R model [41]. Perceived team support, as an organizational resource, plays an extrinsic motivational role by helping employees to achieve their work goals (see [18]). In other words, when employees find their work environment supportive, they will ensure that their task is successfully implemented, making them willing to devote their efforts and abilities to perform their work tasks. 

Regarding the antecedents of technology acceptance, this study’s empirical findings support the hypotheses concerning the effects of perceived usefulness (H2a) and perceived ease of use (H2b) on users’ technology acceptance. In line with TAM literature [24,61,62,70], our study results show strong relationships between these two key user perceptions and technology acceptance. In other words, the extent to which employees who are working remotely using newly implemented technologies find a tool easy to use and helpful in accomplishing job tasks affects whether they will accept and use it. 

In addition to analyzing original TAM constructs, we added virtual job characteristics (i.e., perceived team support and mental load) as external factors to improve the model’s explanatory power. Among these two concepts, only mental load showed a weak negative significant relationship with users’ technology acceptance during remote working. Therefore, H3a was rejected while H3b was supported. Some studies found a similar result regarding the relationship between organizational support and technology acceptance [61,95]. This result is also in line with one of the UTAUT hypotheses (i.e., H4a) test results that assumed that facilitating conditions do not significantly influence behavioral intention [72]. This nonsignificant relationship might result from the sudden start of remote working due to the COVID-19 situation. Especially in the university context, neither the organization nor academic employees were ready for this change. At the same time, they had to restructure processes and change working methods to adapt to this sudden new technological change. In this situation, employees investigated new technology to find a similar way to their traditional way of working. In other words, when employees are not ready to change their working conditions, such as method, location, and tools, they evaluate and accept the new condition based on their perceptions and experience of a similar situation. Therefore, other factors like team support do not have a significant role in accepting new technology.

On the other hand, the results showed that the mental load negatively affects technology acceptance. Although the direct effect of mental load on technology acceptance has not been investigated so far, this result agrees with Dang et al.’s [74] findings which suggested that mental load will negatively affect behavioral intention through performance expectancy, effort expectancy, and facilitating conditions. This result supports that mental load in the remote working situation will affect the user’s decision to use technology. In fact, when using a new tool requires more attention and concentration from employees, they may find the tool useless, and so refuse it. 

This study also supports the idea that remote workers’ acceptance of technology affects their work engagement (H4). This finding approves the findings of Molino et al.’s study [31] and aligns with the literature on technology use and well-being that emphasizes a positive relationship between these two constructs (e.g., [14,21,28,78]). This result confirms our argument regarding the positive role of technology as a job resource in increasing the well-being of employees working from home. In other words, after the urgent technological change in organizations due to the COVID-19 pandemic, technology as a job resource supported employees by providing facilities to perform their work beyond the traditional physical work environment, which motivated them to be engaged in their work activities. Therefore, when employees perceive that their jobs are supported and enriched by the new useful digital technologies, according to the effort recovery model [96], their willingness to dedicate their efforts and abilities to the work task will increase, which will lead to work engagement [18].

In addition, the mediating role of technology acceptance between virtual job characteristics (H5) and work engagement is supported. Although the mediating role of technology acceptance in relationships between work engagement and perceived team support and mental load has not been investigated in previous studies, this result aligns with the JD-R model. In other words, technology use acted as a job resource for remote workers who were experiencing mental load after starting to work from home; it reduced the possible adverse effects of mental load and increased its positive effects on work engagement, resulting in increased work engagement. Also, in line with the motivational process of the JD-R model, technology acceptance multiplied the effect of perceived team support on work engagement by encouraging employees to achieve their job tasks [41]. 

Finally, this study supports that technology acceptance partially mediates the relationship between technology-related perceptions and work engagement (H6). This result shows that when remote workers using new technologies found these technologies easy to use and useful, they became more inclined to reuse them and accept them as a job resource. Therefore, as mentioned earlier, technology acceptance has increased employees’ motivation and engagement in work by helping them to accomplish their goals. 

### 4.2. Managerial Implications 

Our study has significant implications for managers, especially those working in universities, because they need to know what factors have influenced employees’ work-related well-being while working remotely using newly introduced technologies during the COVID-19 situation. Hence, managers probably need to apply different strategies to improve employees’ well-being.

In particular, our study suggests that when it comes to implementing new technologies and changing traditional working methods, managers might focus not only on job resources (i.e., team support) and challenging job demands (i.e., mental load), but on influencing members’ technology acceptance to enhance their work-related well-being further. For example, our findings show that introducing more promising digital technologies increases members’ positive perceptions of those technologies and enhances their technology acceptance level, which facilitates improvement of their work engagement while working in remote mode.

Next, our results show that although both job characteristics and technology acceptance significantly affected employees’ work-related well-being, perceived team support was the strongest determinant of employees’ well-being during this crisis. This result suggests valuable guidance for managers in identifying needs and providing support for employees. In other words, the findings show that a supportive work environment motivated employees to accomplish their work tasks when they had to work from home and re-organize all their working methods due to this sudden technological change.

Finally, the results suggest that when organizations introduce new technology, employees’ perception of the technology may have a more substantial impact on their intention to use it than job characteristics, although both are important. Therefore, managers might focus on introducing appropriate technologies in urgent situations where job resources are limited and job demands increase. For example, by recognizing employees’ needs, managers might evaluate different technologies and introduce more user-friendly and useful technologies, leading to more favorable perceptions of the technology and, in turn, fostering the intention to use the technology.

### 4.3. Limitations and Future Research

One limitation of our research is the use of cross-sectional data to predict the work-related well-being of employees. Therefore, establishing causality relations between variables in this study is not permitted [97]. Although our research is one of the first studies conducted since the onset of the COVID-19 lockdown in Norway, to increase the study’s accuracy, future research should consider a longitudinal study design and explore the long-term effects of technology implementation on employees’ work-related well-being during this crisis. 

The second limitation of this study is focusing only on one particular employee group–academic employees at universities. The work-related well-being determinants we examined may differ in other types of organizations. Therefore, a valuable direction for future research might be to replicate this study in other contexts to improve the generalizability of the findings and gain richer insights. For example, our study revealed a positive relationship between mental load and work engagement that may result from academic employees’ personality characteristics. In other words, academic employees might be more interested in challenging demands than others. In addition, most researchers have been working with many national and international research teams for years, which has required them to use technologies for online collaboration. Furthermore, a flexible work environment at universities has allowed academic staff to work remotely when needed, even before the COVID-19 crisis. Therefore, university employees might be more likely to accept technology or remote work mode than employees working in other types of organizations. 

Thirdly, the current study aimed not to benchmark the included concepts but to assess relations and the theoretical network between concepts. Hence, based on the limited response rate (23.6%), some carefulness should be considered before benchmarking this study results with other samples in different settings.

Another limitation of this study is that we found a relatively low correlation between mental load and technology acceptance. Also, we did not find any association between perceived team support and technology acceptance. Therefore, we suggest exploring the possible role of other job demands or resources in employees’ technology acceptance.

Finally, this study does not represent a complete analysis of the factors influencing employees’ work well-being during the COVID-19 pandemic. There could be other variables impacting this study’s relationships as mediators or moderators. For example, this study has only focused on testing direct and indirect determinants of employees’ well-being; an interesting direction for future research is therefore to investigate the moderating role of some work-related constructs, especially job resources and demands, in the relationship between research variables.

## 5. Conclusions

The present study integrated the JD-R model and TAM to explain the effects of job characteristics, technology-related perceptions, and technology acceptance on the work-related well-being of employees working remotely due to the COVID-19 crisis. Findings indicated that: (i) mental load and perceived support, as two virtual job characteristics, positively predicted employees’ work engagement; (ii) perceived usefulness (PU) and perceived ease of use (PEOU) were positively associated with technology acceptance, while mental load negatively affected it; (iii) technology acceptance was positively associated with work engagement; and (iv) technology acceptance mediated the relationships of virtual job characteristics and technology-related perceptions with work engagement. These findings show that as well as the job characteristics, acceptance of new technologies by employees who have to work from home due to COVID-19 restrictions can play a critical role in their work-related well-being. In other words, these results suggest that although work design is an essential issue for organizations’ leaders, it is vital that they are aware of the features of the technologies, providing the most useful tools for employees who are using them as the only available job resource for communicating and carrying out work activities. 

Importantly, our findings also appear to be helpful beyond the pandemic context and guide organizations regarding flexible work modes even after the COVID-19 pandemic. For example, our research shows that remote workers can also be motivated and engaged in their work if they have access to such necessary resources as technologies and organizational support. In other words, findings reveal that having a flexible work environment is not a problem as long as these necessary resources provide high-quality remote work. Furthermore, this might render most organizations thinking about a more flexible work mode and provide guidance for their future to implement adequate and user-friendly technologies for remote workers, possibly leading to increased work-related well-being.

## Figures and Tables

**Table 1:** Sample characteristics.

	Total	*N*	%
Gender	Male	263	44
	Female	330	56
Age	20–29	35	6
	30–39	118	20
	40–49	179	30
	50–59	149	24
	Over 60 years old	112	20
University	UiS	276	45
	Nord	115	19
	HVL	219	36
Tenure	0–5 years	265	45
	6–10 years	124	21
	11–15 years	66	11
	16–20 years	57	10
	21–25 years	42	7
	Over 26 years	37	6
Education	Bachelor	10	2
	Master	267	45
	PhD	312	53
Main task	Only teaching	67	11
	Only research	94	16
	Both teaching and research	418	70
	Other tasks, more than 30%	22	4
Employment type	Full-time	525	89
	Part-time	67	11

**Table 2:** Internal consistency and convergent validity of the constructs.

Dimension	Items No.	Item	Cronbach’s Alpha	CR	AVE	Factor Loadings
Work engagement			0.75	0.76	0.53	
	WE1	At my work, I feel bursting with energy.				0.65
	WE2	I am enthusiastic about my job.				0.87
	WE3	I am immersed in my work.				0.64
Technology acceptance			0.81	0.82	0.62	
	TA1	I am satisfied with the performance of these digital tools.				0.77
	TA2	I am pleased with the experience of using these digital tools.				0.88
	TA3	Using these digital tools has helped me to improve my work.				0.70
Perceived ease of use			0.87	0.87	0.64	
	PEOU1	My interaction with these digital tools is clear and understandable.				0.74
	PEOU2	Interacting with these digital tools does not require a lot of mental effort.				0.73
	PEOU3	I find these digital tools easy to use.				0.87
	PEOU4	I find it easy to get these digital tools to do what I want them to do.				0.87
Perceived usefulness			0.92	0.92	0.81	
	PU1	Using these digital tools will improve my performance in my job.				0.82
	PU2	Using these digital tools will improve my productivity in my job.				0.96
	PU3	Using these digital tools will enhance my effectiveness in my job.				0.93
Perceived team Support			0.87	0.87	0.64	
	PTS1	The department cares about my general satisfaction at work.				0.88
	PTS2	Even if I did the best job possible, the department would fail to notice.				0.65
	PTS3	The department really cares about my well-being.				0.87
	PTS4	The department takes pride in my accomplishments at work.				0.77
Mental load			0.79	0.74	0.50	
	ML1	My work demands much concentration.				0.71
	ML2	My work requires continual thought.				0.83
	ML3	I have to give continuous attention to my work.				0.70
	ML4	My work requires a great deal of carefulness.				0.55

**Table 3:** Descriptive statistics, correlations, and discriminant validity test results.

Variable	M	SD	1	2	3	4	5	6	7	8
1. Work engagement	3.77	0.72	0.728							
2. Technology acceptance	3.43	0.86	0.18 **	0.787						
3. Perceived ease of use	3.75	0.93	0.15 **	0.56 **	0.805					
4. Perceived usefulness	3.13	1.11	0.14 **	0.67 **	0.46 **	0.902				
5. Perceived team support	3.53	0.94	0.27 **	0.15 **	0.11 **	0.12 **	0.801			
6. Mental load	4.40	0.59	0.14 **	−0.09 *	−0.02	−0.07	−0.02	0.710		
7. Gender ^a^	-	-	0.095 *	0.07	0.01	0.09 *	0.03	0.11 **	-	
8. Age ^a^	-	-	0.04	−0.11 **	−0.28 **	−0.13 **	−0.01	0.02	−0.006	-

Note: Square root of AVE appears on the diagonal. * *p* < 0.05; ** *p* < 0.01. ^a^ Gender: Male = 1, Female = 2; Age: 20–29 = 1, 30–39 = 2, 40–49 = 3, 50–59 = 4, over 60 = 5.

**Table 4:** Indirect effects using bootstrapping (2000 replications).

Indirect Effect	Est.	SE	*p*	CI 95%
Mental load → Technology acceptance → Work engagement	−0.020	0.011	0.012	(−0.044, −0.006)
Perceived Support → Technology acceptance → Work engagement	0.008	0.005	0.036	(0.001, 0.019)
Perceived ease of use → Technology acceptance → Work engagement	0.074	0.023	0.000	(0.041, 0.118)
Perceived usefulness → Technology acceptance → Work engagement	0.042	0.013	0.001	(0.022, 0.067)

## Data Availability

The data presented in this study are available on request from the corresponding author.
